# Non-endodontic periapical lesions clinically diagnosed as endodontic periapical lesions: A retrospective study over 15 years

**DOI:** 10.4317/jced.57957

**Published:** 2021-06-01

**Authors:** Theerachai Kosanwat, Sopee Poomsawat, Jira Kitisubkanchana

**Affiliations:** 1 DDS, Department of Oral and Maxillofacial Pathology, Faculty of Dentistry, Mahidol University, Bangkok, Thailand; 2 DDS, MSc, PhD, Associate Professor, Department of Oral and Maxillofacial Pathology, Faculty of Dentistry, Mahidol University, Bangkok, Thailand; 3 DDS, PhD, Associate Professor, Department of Oral and Maxillofacial Radiology, Faculty of Dentistry, Mahidol University, Bangkok, Thailand

## Abstract

**Background:**

This study aimed to provide the frequency and demographic data of non-endodontic periapical lesions clinically misdiagnosed as endodontic periapical lesions from a Southeast Asian population over a 15-year period.

**Material and Methods:**

A retrospective study was conducted from departmental archives between 2005 and 2019. Cases clinically diagnosed as endodontic periapical lesions were retrieved. Then, cases with a histopathological diagnosis of non-endodontic periapical lesion were selected. Demographic data of non-endodontic periapical lesions were recorded. Radiographic features of cases with available radiographs were analyzed.

**Results:**

Of 1,566 cases clinically diagnosed as endodontic periapical lesion, 157 cases received a histopathological diagnosis of non-endodontic origin. Eighteen different histopathological diagnoses were identified. The most frequent lesion was dentigerous cyst (n= 51, 32.48%) followed by odontogenic keratocyst (n=31, 19.75%), nasopalatine duct cyst (n=18, 11.46%) and ameloblastoma (n=15, 9.56%). Three cases of malignant tumors, including adenoid cystic carcinoma, mucoepidermoid carcinoma, and metastatic papillary thyroid carcinoma were observed.

**Conclusions:**

Non-endodontic periapical lesions constituted 10.03% of cases clinically diagnosed as endodontic periapical lesions. Histopathological examinations of non-endodontic periapical lesions revealed a variety of lesions ranging from foreign body reaction, cysts, fibro-osseous lesions, benign tumors and primary or metastatic malignant tumors. Of clinical significance is that some non-endodontic periapical lesions had different treatment modalities and prognoses compared with endodontic lesions. Therefore, dentists must be aware that periapical radiolucent lesions are not always a consequence of pulpal necrosis.

** Key words:**Ameloblastoma, dentigerous cyst, endodontic periapical lesions, non-endodontic periapical lesions, odontogenic keratocyst.

## Introduction

The majority of periapical radiolucent lesions are a consequence of dental pulp necrosis. These lesions are designated as endodontic periapical lesions (1-4). Histopathological diagnoses of endodontic periapical lesions with radiolucency can be categorized as radicular cyst, periapical granuloma, and periapical abscess (1-4). Several studies have shown that these endodontic periapical lesions constitute 73.0 to 99.4% of periapical radiolucent lesions. Notably, the percentage of these lesions is very disparate in years ranging from 2 to 65 years (1-3,5-10).

The other group of periapical radiolucent lesions is non-endodontic periapical lesions. They are less common than endodontic periapical lesions. Previous studies have reported that non-endodontic periapical lesions consisted of various types of diseases such as cysts, benign tumors, malignant tumors, fibro-osseous lesions, and bacterial or fungal infections (1-3,5,6,11). Therefore, treatments of non-endodontic periapical lesions differ depending on the types of lesions. For example, medication is used to treat microorganism infections whereas enucleation is designated for cystic lesion. Conservative surgical excision is recommended to treat benign tumors whereas radical excision is used to treat malignant tumors (12). Because treatments between endodontic and non-endodontic periapical lesions may greatly differ, recognizing non-endodontic periapical lesions is of clinical significance. Importantly, misdiagnoses of non-endodontic periapical lesions as endodontic periapical lesions have been previously reported and some are the cause of failure in root canal treatment (9,11,13-16). Nobuhara *et al*. (9) studied 150 periradicular specimens obtained from teeth refractory to non-surgical endodontic treatment. Although histopathological diagnoses of most failed cases comprised endodontic lesions, a few were non-endodontic lesions including lateral periodontal cyst, foreign body reaction, myxomatous tissue and calcified tissue fragments. Gondak *et al*. (13) reported five cases of unicystic ameloblastoma, initially diagnosed as apical periodontitis. Two of five cases showed a lack of radiographic regression of the lesions after endodontic treatment. In a series of central giant cell granulomas (CGCGs), 16/79 cases were associated with a tooth having a history of pulp necrosis. These data suggest that CGCGs can be misdiagnosed as endodontic lesions particularly among lesions associated with teeth with necrotic pulps (15).

According to the English-language literature, a few studies have investigated non-endodontic periapical lesions that were clinically misdiagnosed as endodontic periapical lesions (1-3,5-8,17). Among these studies, Huang’s study (1) is the only report from an Asian population. To the best of our knowledge, the study of non-endodontic periapical lesions from Southeast Asia has never been reported. Thus, the present study aimed to provide the frequency and demographic data of non-endodontic periapical lesions clinically diagnosed as endodontic periapical lesions from specimens diagnosed in our institute with 15 years’ experience. Additionally, radiographic features of non-endodontic periapical lesions with available radiographs were studied and analyzed.

## Material and Methods

The present study was approved by the institutional ethics committee (COA.NO.MU-DT/PY-IRB 2019/020.2304) and was conducted according to the guidelines of the Declaration of Helsinki.

Biopsy records, requested from dentists outside our institute and our own institute, submitted for histopathological examination at the Department of Oral and Maxillofacial Pathology, were reviewed. Records between 2005 and 2019 were examined. All cases with clinical diagnoses of endodontic periapical lesions including radicular cyst, periapical granuloma, periapical abscess, asymptomatic apical periodontitis, and apical scar were retrieved. The clinical diagnoses of these cases were made by oral and maxillofacial surgeons, endodontists and general practitioners who submitted the specimens. Before making the clinical diagnoses, clinical and radiographic examinations were considered.

The histopathological diagnoses of these retrieved cases were reviewed. All histopathological diagnoses were made by board certified oral pathologists. Then, cases that were histopathologically diagnosed as non-endodontic periapical lesions were selected. Demographic data including age, sex, and location of all non-endodontic periapical lesions were recorded.

To better understand the characteristics of cases clinically misdiagnosed as endodontic periapical lesions, we further evaluated the radiographic features of these cases. However, we could analyze only radiographs of cases from our own institute as we could not retrieve radiographs of cases outside our institute. All radiographs were reviewed by TK (resident in oral diagnostic science). Before analyzing, radiographic interpretation was calibrated by JK (board certified oral and maxillofacial radiologist). Radiographic features of non-endodontic periapical lesions were interpreted from available radiographs both in digital and analog systems. Radiographic features of lesions were recorded in terms of margin and shape of the lesions. Lesions that could be identified regarding the limits of the lesion with confidence were described as “well-defined margin” while lesions that were difficult to indicate an exact delineation around the lesion were designated as “ill-defined margin”. A shape of each lesion was classified as unilocular shape when the lesion showed a circular/fluid-filled shape or multilocular shape when the septa appeared to divide the internal structure of the lesion into at least two compartments. All data were descriptively analyzed.

## Results

A total of 1,566 cases were clinically diagnosed as endodontic periapical lesions after reviewing the requested biopsy records between 2005 and 2019. Of the 1,566 cases, 157 cases (10.03%) received a histopathological diagnosis of non-endodontic periapical entities.

Demographic data of non-endodontic periapical lesions revealed that 89 patients were male and 68 patients were female with a male to female ratio of 1.3:1. There were 82 cases at the maxilla and 75 cases at the mandible.

Histopathologically, 18 different histopathological diagnoses were identified (Table 1). Interestingly, a wide variety of histopathological diagnoses such as cysts, benign tumors, malignant tumors, and fibro-osseous lesions were discovered. The most frequent non-endodontic periapical lesion was dentigerous cyst (n= 51, 32.48%) followed by odontogenic keratocyst (OKC) (n=31, 19.75%), nasopalatine duct cyst (n=18, 11.46%) and ameloblastoma (n=15, 9.56%). Of 51 cases of dentigerous cyst, 16 cases were associated with unerupted mandibular third molar followed by unerupted mesiodens (12 cases), unerupted maxillary canine (7 cases), and other teeth (16 cases). Nine cases of ameloblastoma were classified as unicystic type and the remaining six cases were classified as conventional ameloblastoma.

**Table 1:**
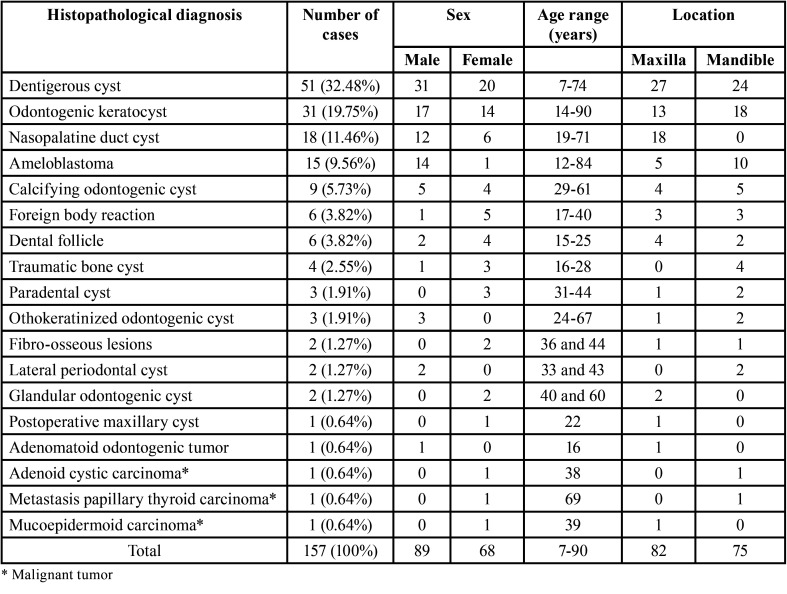
Histopathological diagnosis, number, and demographic data of non-endodontic periapical lesions.

**Figure 1:**
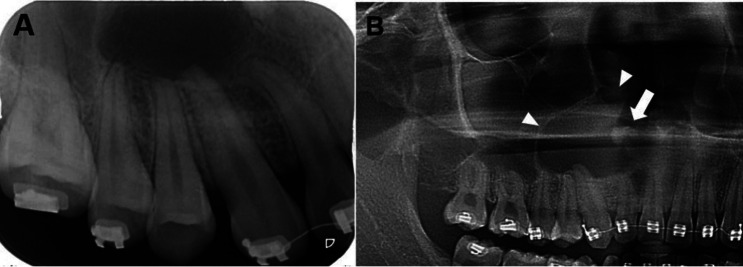
Radiographs of a dentigerous cyst. A. A periapical radiograph reveals a well-defined radiolucent lesion associated with apical regions of upper right lateral incisor to the second premolar, leading to the misdiagnosis of endodontic lesion. B. A cropped panoramic radiograph of the same patient reveals entire part of the lesion which is associated with an impacted supernumerary tooth (arrow). The floor of the right maxillary sinus and nasal cavity are elevated by the lesion (arrowheads).

**Figure 2:**
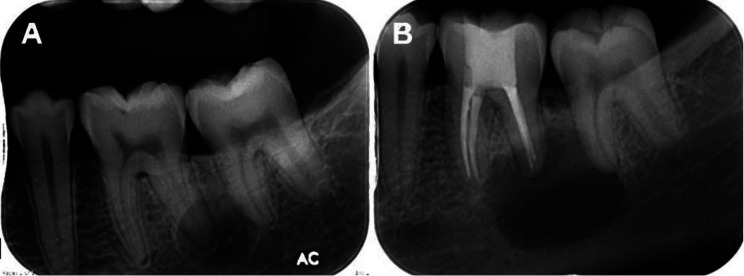
Periapical radiographs of an odontogenic keratocyst. A. A well-defined unilocular radiolucent lesion with corticated border is superimposed over the distal root of the lower left first molar. A thin radiolucent line of the periodontal ligament space of this root is clearly observed. B. The same lesion became larger despite root canal treatment for one year.

**Figure 3:**
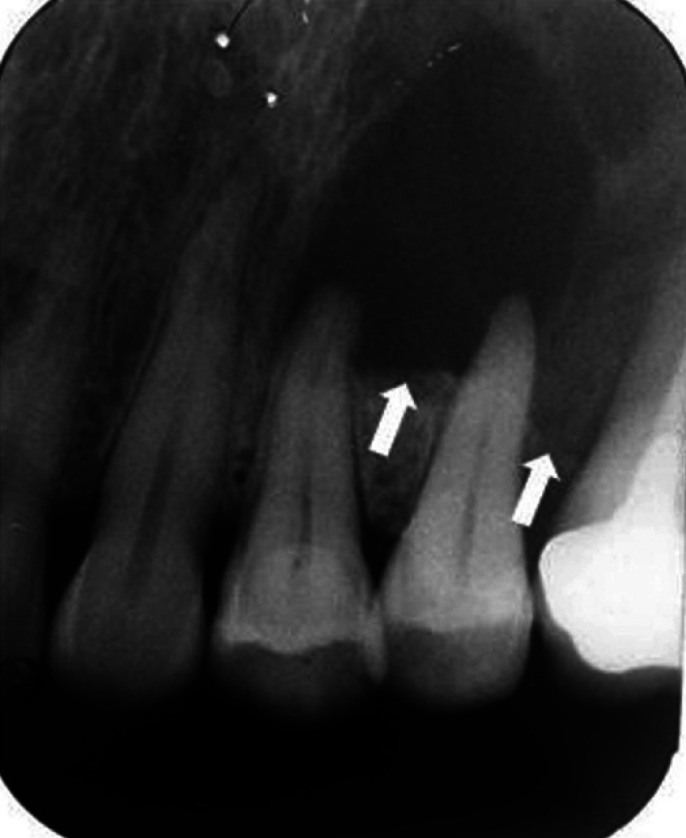
A periapical radiograph of a central mucoepidermoid carcinoma shows ill-defined radiolucent area at the left posterior maxillary region. Inferior border of the lesion is indicated by arrows. The superior and posterior borders of the lesion cannot be revealed on this periapical radiograph.

Of 157 cases of non-endodontic periapical lesions, only 78 cases were derived from our institute. Therefore, we could only analyze radiographs of these 78 cases. Radiographic features of these cases are summarized in Table 2. Of 51 dentigerous cysts, radiographs of 24 cases were available. All lesions showed well-defined unilocular radiolucency associated with unerupted teeth. However, these lesions were also frequently associated with apex of adjacent erupted tooth/teeth. An example of a dentigerous cyst clinically misdiagnosed as radicular cyst is shown in Figure 1. The initial diagnosis of radicular cyst was made from findings on a periapical image which failed to show the entire lesion. Before performing an excisional operation, a panoramic radiograph was taken, revealing the lesion was associated with an impacted supernumerary tooth. Of 31 OKCs, radiographs of 14 cases were available. Most OKCs (12/14) demonstrated a well-defined unilocular radiolucency (Fig. 2) while two cases presented as multilocular radiolucency with well-defined border. Radiographs of 10 out of 15 cases of ameloblastoma were available. They revealed that 80% (8/10) were well-defined unilocular radiolucency and 20% (2/10) were well-defined multilocular radiolucency. With regard to three cases of malignancy, only one periapical radiograph of mucoepidermoid carcinoma was available. The lesion caused bone erosion leading to an ill-defined border. The area of bone destruction was far superior and posterior beyond the scope of this radiograph (Fig. 3).

**Table 2:**
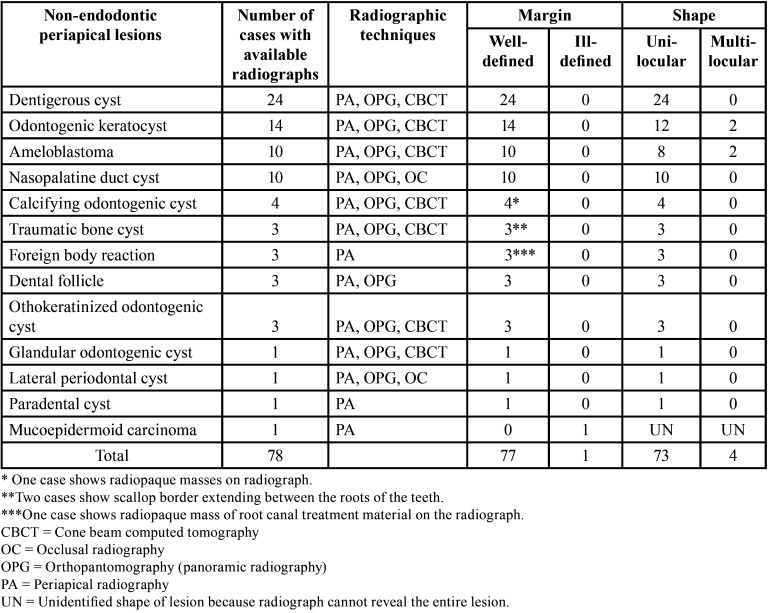
Radiographic features from available radiographs of non-endodontic periapical lesions in the present study.

## Discussion

Our study showed that 10.03% of lesions clinically diagnosed as endodontic periapical lesions were histopathologically rendered as non-endodontic periapical lesions. The frequencies of non-endodontic periapical cases in our study were higher than those of previous studies, reporting from 0.64 to 4.22% of periapical radiolucent lesions as shown in Table 3 (1-3,5-7). The male to female ratio in our study was 1.3:1. The slight male predilection of our study is consistent with those of Huang *et al*. (1) and Kontogiannis *et al*. (5) but in contrast to other studies (2,3,7) in which females were predominant. The frequencies of cases occurring in the maxilla compared with those in the mandible did not differ much. Approximately 52% of non-endodontic lesions occurred at the maxilla and 48% occurred at the mandible.

**Table 3:**
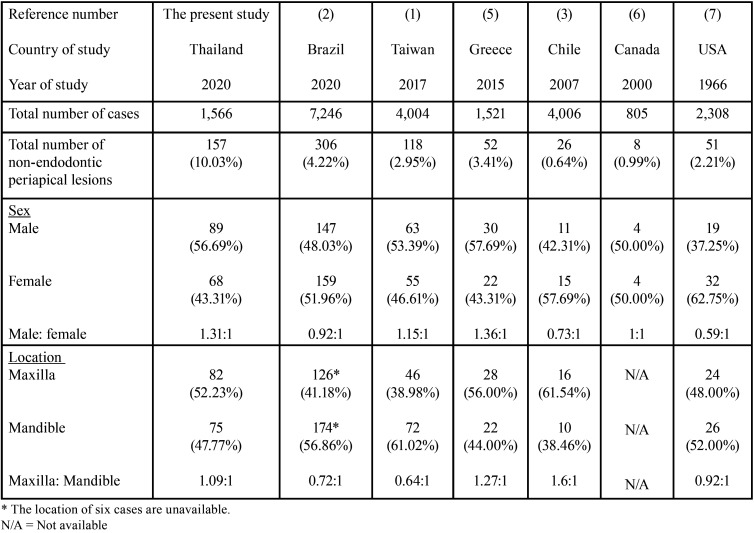
Comparisons of number, sex, and location of non-endodontic periapical lesions in the present study with those of previous studies.

Eighteen different histopathological diagnoses were identified in our study. The result is consistent with previous studies reporting 7 to 28 different histopathological diagnoses (1-3,5,6). A wide variety of diseases of non-endodontic periapical lesions including cysts, benign tumors, malignant tumors, and fibro-osseous lesions was observed in our study. Similar to the results from four previous studies (1-3,5), cystic lesions were the most common non-endodontic periapical lesion in our study. Based on our study, the most common non-endodontic periapical lesion was dentigerous cyst followed by OKC. This data is in line with four previous studies showing that OKC was the most frequent lesion (1-3,5), and two studies (1,2) reporting that dentigerous cyst was the second and the third most common lesion. Three cases of malignancy were found in our study. This result is comparable to those of Kuc *et al*. (6) and Kontogiannis *et al*. (5) reporting one case each of malignancy. However, Vieira *et al*. (2) and Huang *et al*. (1) reported eleven and nine cases of malignant lesions, respectively.

Dentigerous cyst was the most frequent lesion found in our study. It constituted 32.48% of non-endodontic cases. The number of dentigerous cysts in this study was higher than three previous studies, ranging from 3.85 to 15.68% (1,2,5). The other two studies did not find dentigerous cyst in their series (3,6). Although dentigerous cysts in our study usually showed well-defined unilocular radiolucency around the crown of impacted tooth, almost all of the lesions involved root apex of an adjacent erupted tooth/teeth. For this reason, clinicians diagnosed the lesions as radicular cyst of the adjacent erupted tooth instead of dentigerous cyst. To make a correct diagnosis, careful clinical examination of the involved teeth is helpful. For example, when the lesion involves the apex of an erupted tooth without carious lesion, a diagnosis of radicular cyst is unlikely. By contrast, a large carious lesion of an adjacent erupted tooth may suggest a diagnosis of radicular cyst rather than dentigerous cyst. The electric pulp test (EPT) is an additional tool to make a diagnosis of such lesion. The negative result for EPT and deep carious lesion suggest that the lesion is of endodontic origin. Furthermore, clinical misdiagnosis may be easily encountered when the radiograph does not reveal the entire lesion as shown in Figure 1A. Importantly, clinicians should be aware that the essential first step for radiographic interpretation is that the radiograph should completely display the entire lesion of interest.

Previous studies have found that OKC was the most common non-endodontic periapical lesion comprising 32.20 to 42.31% of cases (1-3,5). In our study, OKC was the second most common lesion totaling 19.75% of non-endodontic periapical lesions. From 14 cases of OKC with available radiographs, 2 cases showed well-defined multilocular radiolucency. These radiographic features are helpful for clinicians to distinguish OKC from endodontic periapical lesions. Nevertheless, more than 80% of OKC (12/14 cases) demonstrated well-defined unilocular radiolucency at the periapical region as shown in Figure 2. This radiographic feature is difficult for clinicians to make a clinical diagnosis of OKC, particularly when the lesion associated with a deep carious tooth or endodontically treated tooth. OKC with unilocular radiolucency, associated with an endodontically treated tooth, could be misdiagnosed as radicular cyst or periapical granuloma. Garlock *et al*. (14) reported that this kind of misdiagnosis comprised 5% of OKCs in their study. Owing to aggressive behavior and tendency for recurrences of OKC (14), submitting all tissues from periapical lesions for histopathological diagnosis is recommended. In case that microscopic examination has not been performed, periodic follow-up is suggested. When the lesion becomes larger or does not improve after initial treatment, an excisional biopsy for a definitive diagnosis should be performed.

Similar to Huang *et al*. (1) and Vieira *et al*. (2), ameloblastoma was the most common benign tumor observed in our study accounting for 9.56% (15 cases) of non-endodontic periapical lesions. Ortega *et al*. (3) and Kuc *et al*. (6) found no ameloblastoma in their studies, and one case (1.92%) of ameloblastoma was reported from Kontogiannis’s study (5). Among 15 cases of ameloblastoma in the present study, more than one half of cases (9/15) received a histopathological diagnosis of unicystic ameloblastoma. This is not surprising because unilocular radiolucency is the typical radiographic feature of unicystic ameloblastoma. Thus, a small unicystic ameloblastoma may mimic those of endodontic periapical lesions. Gondak *et al*. (13) reported five cases of unicystic ameloblastoma misdiagnosed as apical periodontitis. They discussed that unicystic ameloblastoma, localized in the periapical region, may be easily misdiagnosed as endodontic periapical lesions (13). Radiographically, two types of radiographic features of ameloblastoma were observed in our study. The majority of ameloblastoma (80%) showed unilocular radiolucency with a well-defined border whereas 20% demonstrated well-defined multilocular radiolucency at the periapical region. Radiographic features of ameloblastoma with well-defined multilocular radiolucency should alert clinicians to make a diagnosis of non-endodontic periapical lesion.

In our study, two malignant salivary gland tumors including adenoid cystic carcinoma and mucoepidermoid carcinoma were found. Compared with previous studies, Vieira *et al*. (2) and Huang *et al*. (1) reported four cases and one case of adenoid cystic carcinoma at the periapical region, respectively. Two cases of mucoepidermoid carcinoma were found from Vieira’s study (2) while other studies did not report mucoepidermoid carcinoma at the periapical area (1,3,5,6). Although central mucoepidermoid carcinoma was rare (18-20), it was the most common salivary gland tumor occurring in the jaw bone (21). Radiographic features of central mucoepidermoid carcinoma can show either unilocular or multilocular radiolucency with well-defined borders. However, some cases of central mucoepidermoid carcinoma demonstrated radiolucency with ill-defined margin (18,22). In our case, the lesion showed ill-defined radiolucency at posterior maxilla in a periapical image. Generally, the extent of the lesion occurring at the posterior maxillary area as in our case might be difficult to evaluate because the lesion may be superimposed with the maxillary sinus. Consultation with oral radiologists is recommended to reach precise radiographic interpretation in such a location. Furthermore, the periapical image cannot display the entire lesion in this case. Therefore, other radiographic techniques revealing the entire lesion such as panoramic and computed tomography images should be performed to evaluate radiographic features before making a clinical diagnosis.

Metastatic malignant tumors to the jaw bone can also mimic radiographic features of endodontic periapical lesions. One case of metastatic carcinoma was reported from Kontogiannis’s study (5). The posterior mandible was the predilection area of metastatic tumors (23-25). Regardless of sex, the most common origins of the metastatic lesions to the jaws derived from the breast, lung, kidney, and adrenal gland, respectively (23,26,27). In our study, one case of metastatic papillary thyroid carcinoma was found. The lesion was an asymptomatic, well-defined unilocular radiolucency associated with the apical area of two teeth. Based on these radiographic features, the clinician diagnosed this lesion as an endodontic periapical lesion. However, this patient reported weight loss of two kilograms in one month and presented enlarged lymph nodes. These signs and symptoms were unusual findings for endodontic periapical lesions. Notably, data from history taking and extraoral examination should be considered to make a correct diagnosis.

Of clinical significance is the misdiagnosis of benign aggressive lesions, such as ameloblastoma and OKC, and malignant tumors as endodontic lesions. This is because the treatments of these lesions are more aggressive than those of endodontic lesions. For example, to reduce recurrence, enucleation and curettage with peripheral ostectomy are recommended to treat OKC (14). As for ameloblastoma, the treatment ranges from simple enucleation and curettage in small lesions to partial mandibulectomy/maxillectomy in large lesions (28). In the case of malignancy, the treatment is radical surgery combined with adjunctive chemotherapy or radiotherapy depending on cancer types (23,25). By contrast, root canal treatment or simple enucleation is sufficient to treat lesions of endodontic origin (4,9,29). To avoid misdiagnosis and achieve accurate treatment; we therefore suggest that clinicians should submit all specimens removed from patients for histopathological investigation.

Failure in endodontic treatment may be associated with non-endodontic periapical lesions (9). From previous studies, subsequent biopsies of periapical lesions with failure in endodontic treatment have been shown to be of non-endodontic origin including OKC (14), ameloblastoma (13), and lateral periodontal cyst (9). Similar to one case of OKC in the present study, the lesion was initially diagnosed as an endodontic lesion and received root canal treatment. However, the size of the lesion increased after one year follow-up. Then, a biopsy was performed to identify the true nature of the lesion and finally, the histopathological diagnosis of OKC was made. Based on this evidence, non-endodontic periapical lesions should be included in the differential diagnosis of periapical lesions that are refractory to endodontic treatment.

## Conclusions

Non-endodontic periapical lesions constituted 10.03% of cases clinically diagnosed as endodontic periapical lesion. Histopathological examinations of non-endodontic periapical lesions revealed a variety of lesions ranging from foreign body reaction, cysts, fibro-osseous lesions, benign tumors and primary or metastatic malignant tumors. Our study found that dentigerous cyst was the most common lesion, followed by OKC. Of clinical significance is that some non-endodontic periapical lesions have different treatment modalities and prognosis compared with those of endodontic lesions. Therefore, dentists must be aware that periapical radiolucent lesions are not always a consequence of pulpal necrosis.
